# Investigating zero transmission of HIV in the MSM population: a UK modelling case study

**DOI:** 10.1186/s13690-023-01178-0

**Published:** 2023-11-20

**Authors:** Katherine Massey, Vittoria Vardanega, Mas Chaponda, Lucy A. Eddowes, Natalie Hearmon

**Affiliations:** 1 Costello Medical, 50/60 Station Road, Cambridge, CB1 2JH UK; 2 Costello Medical, 55 Old Broad Street, London, EC2M 1RX UK; 3 Hamad Medical Corporation, Doha, Qatar

**Keywords:** HIV/AIDS, Zero transmission, Prevention, Public health, HIV policy, PrEP

## Abstract

**Background:**

UNAIDS 90-90-90 goals for HIV have been surpassed in the UK, with focus now moving to ending transmission by 2030. The concept of zero transmission is complex and many factors can influence transmission. We aimed to investigate how the target of zero transmission might be reached in the UK.

**Methods:**

We developed a *de novo* Markov state transition open cohort model of HIV with a 50-year time horizon, which models six key screening, treatment and prevention parameters, including treatment-as-prevention (TasP) and pre-exposure prophylaxis (PrEP). We studied the anticipated HIV epidemic trajectory over time in men who have sex with men (MSM), with and without changing the six key parameters, defining zero transmission as a 60% reduction in incidence compared with 2010 incidence.

**Results:**

Zero transmission in the MSM population was not achieved within the model’s time horizon in our base case scenario, when the six key parameters were set to their 2019 values. Several future scenarios were explored, including a combination approach to preventing HIV transmission through increasing five key parameter values and considering three different TasP values; zero transmission was achieved by 2030 in the scenario where TasP was increased from its current level of 97–99%, avoiding 48,969 new HIV cases over the time horizon and reducing the lifetime risk of acquiring HIV for HIV-negative MSM not using PrEP from 13.65 to 7.53%.

**Conclusions:**

Zero transmission in the UK MSM population can be reached by the target year of 2030 with bold changes to HIV policy. A combination approach such as the UK Government’s ‘Towards Zero’ Action plan, impacting multiple policies and including an increase in TasP, has the potential to achieve meaningful reductions in HIV transmission and meet this ambitious goal.

**Supplementary Information:**

The online version contains supplementary material available at 10.1186/s13690-023-01178-0.

**Table:** 

Text box 1. Contributions to the literature	
• The UK government has set a target to reach zero transmission of HIV by 2030, but few studies explore how the UK might reach this goal and the progress that is being made towards it.	
• This research in MSM, a key group for HIV policy, suggests that the UK is unlikely to reach its zero transmission goal without changes to HIV prevention, screening and treatment policies.	
• Ambitious public health policies that use a combination approach to limit HIV transmission offer the best hope for the UK to reach zero transmission in the MSM population by 2030.	

## Background

The Joint United Nations Programme on HIV/AIDS (UNAIDS) 90-90-90 targets were introduced in 2014 as goals to help end the human immunodeficiency virus (HIV)/acquired immunodeficiency syndrome (AIDS) epidemic by 2020, by ensuring that people living with HIV (PLWH) are aware of their status, receiving antiretroviral therapy (ART) and achieving viral suppression [1]. These targets are being surpassed in several countries, including the UK, Australia, Botswana and Thailand; going forwards, UNAIDS and individual countries are adopting the more ambitious 95-95-95 by 2030 targets and looking towards ending HIV transmission [2–4].

The UK is one of the first countries to target zero transmission by 2030. Until recently, there was no consensus on how zero transmission should be defined. An independent HIV Commission has suggested that the UNAIDS ‘elimination’ definition of < 1 incident infection per 10,000 individuals per year be used as the definition of zero transmission [5].

A proportion of new HIV diagnoses made in the UK each year originate outside the country; in 2021, it was estimated that 9% of men who have sex with men (MSM) living with HIV (MSMLWH) who were born in the UK likely acquired HIV outside of the UK, while an estimated 67% of MSMLWH who were born outside of the UK likely acquired HIV outside of the UK [6, 7]. Even if HIV transmission is eliminated within the UK, new cases are likely to be identified in any given year due to the immigration of undiagnosed PLWH. Countries targeting zero transmission must account for the impact of both domestically and externally acquired HIV cases, as well as how local HIV policy can influence onwards transmission. There are currently no definitions for zero transmission routinely used in policy; though zero transmission logically should mean no transmissions at all, a practical definition of zero transmission may need to take externally acquired HIV into account.

Reducing HIV transmission requires a focus on prevention, screening and treatment policies, linking PLWH to care following diagnosis, [8–10] quickly identifying new cases of HIV [11] and improving access to pre-exposure prophylaxis (PrEP) for at-risk individuals [12]. Typically, efforts are concentrated in groups in which HIV prevalence and transmission are relatively high. In the UK, MSM represent the group in which most new diagnoses are made each year; [6] reducing HIV transmission in this group is likely to have a substantial impact on the HIV epidemic overall.

In December 2021, the UK Government released a ‘Towards Zero’ HIV Action Plan, setting out their plans and policies to reach the target of zero transmission of HIV in England. The plan aims to reduce the number of people first diagnosed with HIV in England to < 600 by 2025 and outlines objectives to ensure access and uptake of HIV prevention programmes, invest in PrEP, scale up HIV testing and improve rapid access to treatment and retention in care. Though recent studies of HIV transmission and prevalence in England and the UK have investigated how zero transmission can be defined and current progress towards this goal, [13, 14] there are no studies that explore how changes to UK policy to support a combination approach to preventing HIV transmission might influence the trajectory of the HIV epidemic.

To investigate how zero transmission goals may be approached, a *de novo* HIV transmission model with a UK perspective was developed. This study utilised the model to investigate whether the approach outlined in the ‘Towards Zero’ action plan could allow zero transmission to be reached in the UK MSM population. The study also investigated the screening, treatment and prevention factors that are most important for achieving zero transmission in MSM by 2030.

## Methods

Full details of the model are found in the supplementary appendix.

### Model structure and inputs

A Markov state transition open cohort model with a cycle length of three months and a maximum time horizon of 50 years was developed; the cycle length was selected to align with the frequency of PrEP surveillance screening and standard times to initiation of ART in the UK [15]. The model start year is 2020. Model inputs were chosen to reflect the UK MSM population.

**Fig. 1:**
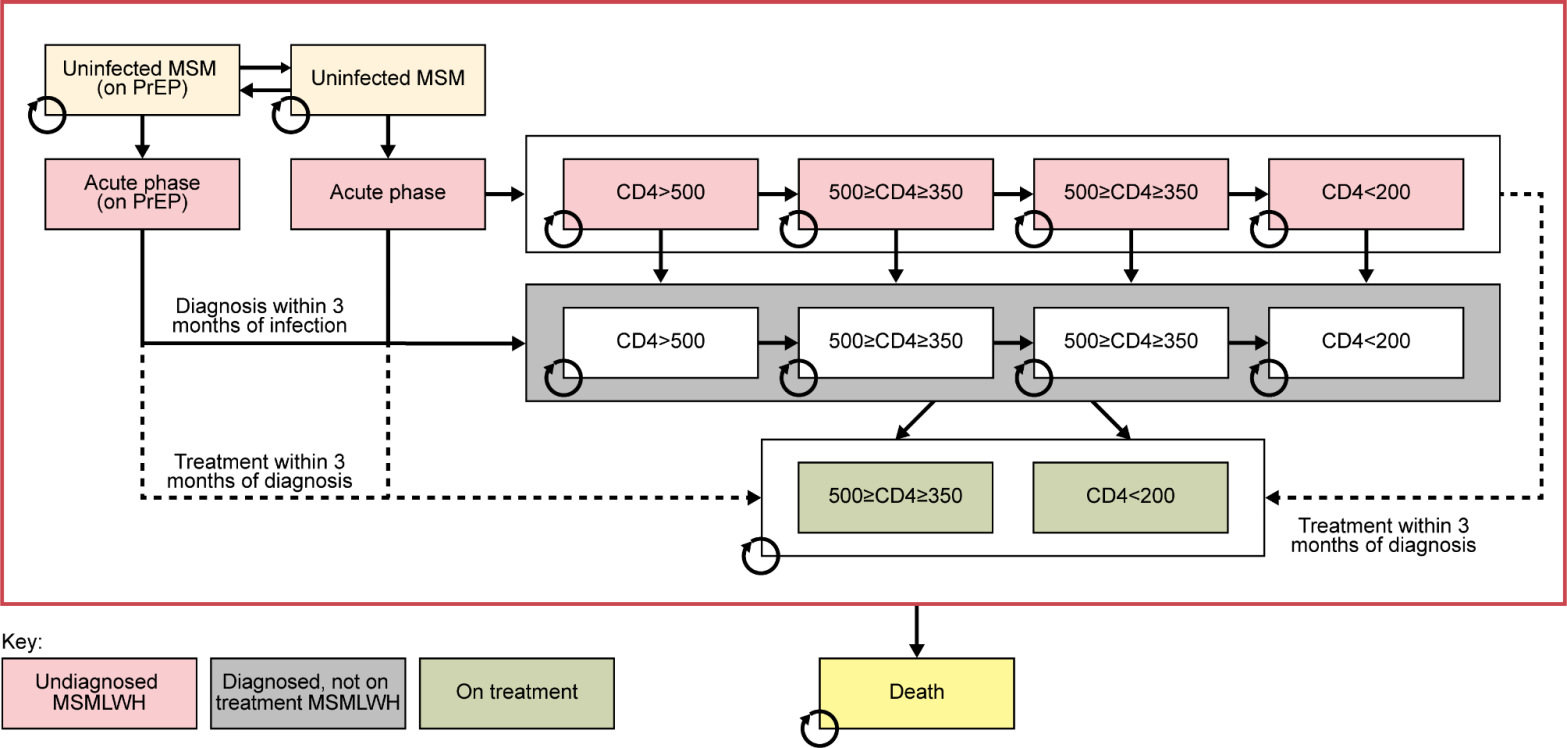
Zero transmission model structure. CD4 counts are in cells/µL. Individuals may transition between health states in the directions of the arrows; transition probabilities are calculated from model inputs. Individuals in all health states have a risk of death; this risk is modified by the average age of the population in question (MSM or MSMLWH) and their disease state. MSM: men who have sex with men; MSMLWH: men who have sex with men living with HIV; PrEP: pre-exposure prophylaxis

Fifteen different health states are used to model MSMLWH and HIV-negative MSM, whether MSMLWH are diagnosed and/or on treatment (affecting disease progression) and whether HIV-negative individuals are using PrEP (affecting their probability of acquiring HIV) (Fig. 1). MSMLWH (diagnosed and undiagnosed) not on treatment are distributed across four CD4 cell count-related health states. Individuals move through the model as shown in Fig. 1, and individuals in all health states experience a probability of dying based on the health state they occupy. The initial population distribution across the health states is taken from 2019 Public Health England (PHE; now UK Health Security Agency [HSA]) data (Table 1) [6, 16].

**Table 1:** Key population and epidemiology inputs, MSM population

Input	Value	Source
Initial population, N	621,210	BASHH 2020, [17] ONS 2018 [18]
MSMLWH population, % (n)	8.097 (50,299)	Public Health England 2020 [16]
Undiagnosed MSMLWH, % (n)	5.77 (2,902)	Public Health England 2020 [16]
Diagnosed MSMLWH, % (n)	94.23 (47,397)	Public Health England 2020 [16]
PrEP users, % of HIV-negative population (n)	4.326 (24,698)	Public Health England 2020, [16] Expert Opinion
Efficacy of PrEP, %	86.00	Cambiano et al., 2018 [19]
Treated population, % of diagnosed population (n)	98.44 (46,658)	Public Health England, 2020 [16]
MSMLWH who are virologically suppressed, % of treated population (n)	97.00 (45,258)	Public Health England, 2020 [16]
Initial health state distribution, undiagnosed population, % (n)		
CD4 > 500	45.80 (1,329)	Public Health England, 2020 [16]
500 ≥ CD4 ≥ 350	19.06 (553)	Public Health England, 2020 [16]
350 > CD4 > 200	1.04 (30)	Public Health England, 2020 [16]
CD4 ≤ 200	34.10 (990)	Public Health England, 2020 [16]
Initial health state distribution, not on treatment population, % (n)		
CD4 > 500	45.80 (339)	Public Health England, 2020 [16]
500 ≥ CD4 ≥ 350	19.06 (141)	Public Health England, 2020 [16]
350 > CD4 ≥ 200	1.04 (8)	Public Health England, 2020 [16]
CD4 < 200	34.10 (252)	Public Health England, 2020 [16]

**Footnotes**: CD4 counts are in cells/µL. BASHH: British Association for Sexual Health and HIV; MSM: men who have sex with men; MSMLWH: men who have sex with men living with HIV; ONS: Office for National Statistics; PrEP: pre-exposure prophylaxis

HIV transmission is calculated using an estimate for the basic reproduction number (the number of new HIV cases generated by one MSMLWH per cycle, if the entire MSM population was susceptible to HIV transmission), *β*. Initially, *β* is calculated using historical data; in subsequent model cycles, *β* is calculated using the results of previous model cycles. The number of transmissions occurring in each cycle is then calculated using *β*, accounting for the use and efficacy of PrEP and ART (set to reduce susceptibility to transmission and risk of transmission by 86% and 100%, respectively [see supplementary appendix]).

To model population growth and HIV acquired outside the UK, new MSM enter the population every cycle and are assumed to be distributed across health states as per the initial model cycle (Table 1).

### Model validation

Validation of the model structure, key inputs and assumptions was sought from disease modelling, clinical and UK public health experts during the development of the model. Feedback was incorporated into model development to ensure it reflects current UK clinical practice and that the results are relevant to the public health landscape.

### Interventions

To investigate how key areas of health policy can influence HIV transmission, the model has six key parameters grouped under two policy aims covering prevention, screening and treatment, including treatment-as-prevention (TasP) and PrEP. These parameters, described in Table 2, are implemented on a three-monthly basis, aligning with model cycle length.

**Table 2:** Key model parameters

Policy Aim	Key Parameter	Description of parameter
Decreasing the size of the susceptible population	Annual probability for an HIV-negative individual to start PrEP	Allows the number of individuals taking PrEP to be adjusted through changing the rate of uptake
Reducing the length of time over which MSMLWH can transmit the virus	Proportion of MSMLWH diagnosed within three months of transmission	Models the early diagnosis of MSMLWH and allows individuals to move to ‘on treatment’ health states more quickly
	Annual probability of screening HIV-negative individuals and undiagnosed MSMLWH	Represents the proportion of MSM tested for HIV every year
	Proportion of MSMLWH starting treatment within six months of diagnosis	Represents the proportion of diagnosed MSMLWH starting treatment within six months of receiving a diagnosis
	Proportion of MSMLWH starting treatment within three months of diagnosis	Models the ‘Immediate ART’ policy in which individuals initiate ART within 90 days of diagnosis [10]
	Proportion of MSMLWH using ART who are virologically suppressed	Defines the proportion of individuals who achieve virologic suppression while on treatment. It is assumed that ART is 100% effective and MSMLWH who are virologically suppressed are unable to transmit HIV to others (also known as U = U). This parameter is henceforward referred to as TasP

**Footnotes**: ART: antiretroviral therapy; MSM: men who have sex with men; MSMLWH: men who have sex with men with living HIV; PrEP: pre-exposure prophylaxis; TasP: treatment-as-prevention; U = U: undetectable = untransmittable

### Model definition of zero transmission

This model takes into account the fact that a proportion of new diagnoses each year arise from HIV exposure outside of the UK and therefore cannot be influenced by UK HIV policy. This model uses a pragmatic definition of zero transmission to account for such diagnoses. In this study, ‘reaching zero transmission’ is defined as a 60% reduction in HIV incidence compared with 2010 HIV incidence. This threshold was selected to account for the fact that in 2010 an estimated 33% of HIV diagnoses in MSM resulted from transmissions occurring outside of the UK; [6] a 60% reduction in incidence from 2010 levels therefore represents the point at which almost all domestic transmission has been eliminated. This threshold is aligned with the UNAIDS ‘elimination’ definition of < 1 new infection per 10,000 individuals per year [5]. When running scenarios the model reports the year in which zero transmission, as defined here, will be reached.

### Model scenarios

The model has two options for generating results, both of which were utilised in this study. Using the ‘single parameter estimation’ method, the user inputs the year in which zero transmission should be reached in the model and selects one of the six key parameters (Table 2) to be varied. The model calculates the input value for the selected key parameter required for zero transmission to be reached by the specified year; the remaining five parameters remain at their base case input values. In the model results presented here, the target year for zero transmission is set to 2030 and values for each of the six key parameters required to meet this target are calculated; details are presented in the Results section.

The second approach allows the user to develop scenarios by changing each of the six key parameters in combination. The model then calculates the year in which zero transmission would be reached based on the scenario inputs for each of the six key parameters. The scenarios, including base case and future scenarios, used for this approach are presented in Table 3.

**Table 3:** Base case and future scenario key parameter inputs

Key parameter	Scenario input values^a^						
	Base case	Combination prevention	Combination prevention, 98% TasP	Combination prevention, 99% TasP	Aspirational combination prevention	NHS-commissioned PrEP base case^b^	NHS-commissioned PrEP combination prevention
Rate of PrEP uptake	0.08%^c^	0.25%	0.25%	0.25%	0.45%	0.08%^c^	0.25%
Proportion of MSMLWH diagnosed within three months of transmission	26.00% [4]	40.00%	40.00%	40.00%	40.00%	26.00% [4]	40.00%
Annual probability of having an HIV test	22.35% [4]	40.00%	40.00%	40.00%	50.00%	22.35% [4]	40.00%
Probability of starting treatment within six months of diagnosis	95.17% [31]	98.00%	98.00%	98.00%	98.00%	95.17% [31]	98.00%
Probability of starting treatment within three months of diagnosis	78.00% [6]	90.00%	90.00%	90.00%	95.00%	78.00% [6]	90.00%
TasP	97% [16]	97%	98%	99%	99%	97% [16]	99%

**Footnotes**: ^a^Future scenario input values are implemented as a linear increase from the 2020 base case value to 2024, with the exception of the date of PrEP uptake which is implemented as a linear increase from 2020 to 2022. ^b^In the NHS-commissioned PrEP base case the number of MSM on PrEP in the first year of the model is increased from the model base case value of 24,698 (based on the number of MSM enrolled on the IMPACT trial) [16] to 50,152 (based on the number of MSM reported to be accessing PrEP through NHS specialist SHSs in 2021) [20]. ^c^Assumption based on PrEP Impact trial uptake [21]. All other base case scenario inputs are based on 2019 Public Health England data. HIV: human immunodeficiency virus; MSMLWH: men who have sex with men living with HIV; NHS: National Health Service; PrEP: pre-exposure prophylaxis; TasP: treatment as prevention

### Patient and public involvement

Patients and the public were not involved in this research.

## Results

### Current trajectory of the HIV epidemic in the MSM population

In the base case scenario, representing the current state of the HIV epidemic in the UK and using model inputs representing current prevention, screening and treatment policies (Table 3), zero transmission was not reached in the MSM population within the model time horizon of 50 years (2020–2070), using the incidence reduction definition.

The rate of PrEP uptake in the base case scenario was based on the rate of uptake to the Impact trial [21] and represents a conservative assumption given the lack of available real-world data on PrEP uptake in the UK. As a result, the proportion of MSM using PrEP in the model increased by a small percentage over time; in 2020, 4.90% (27,974 people) of MSM were using PrEP, compared with a predicted 5.04% (30,696 people) in 2070 (Supplementary Fig. 1).

These base case scenario results demonstrated that improvements in prevention, screening and treatment are required for zero transmission to be reached in the UK MSM population within an ambitious timeframe.

### Reaching zero transmission in the MSM population

Both single- and multi-parameter scenarios were explored to investigate how zero transmission could be reached in the UK MSM population. For all parameters, changes were implemented linearly between 2020 and 2024, except for the rate of PrEP uptake, for which changes were implemented linearly between 2020 and 2022 (see supplementary appendix).

#### Single parameter changes and zero transmission

First, the ‘single parameter estimation’ method was used to explore the changes to each of the six key parameters required for zero transmission to be reached by 2030. Only two parameters, rate of PrEP uptake and probability of screening, could be increased sufficiently for zero transmission to be reached by 2030, in the absence of changes to any other parameters. Two parameters, annual rate of PrEP uptake and annual probability of screening for HIV-negative and undiagnosed individuals, could be individually increased to allow zero transmission to be reached by 2030. An increase in the annual rate of PrEP uptake of 9%, from 0.08% to 9.08% (resulting in 52.16% of MSM [323,923 individuals] using PrEP by the end of 2030; Supplementary Fig. 2) was required to reach the target. A similarly substantial increase of 74.5% in the annual probability of screening, from 22.35% in the base case to 96.85%, was required for the transmission target to be reached. It was not possible for zero transmission to be reached by 2030 if the percentage of individuals diagnosed within three months of transmission, the probability of starting treatment within three or six months of diagnosis, or the use of TasP, were increased alone. These results demonstrated that PrEP use and screening rates are significant drivers of model results, but that extremely large and likely unfeasible increases in these parameters alone are required for zero transmission to be reached by 2030.

#### A combination approach to zero transmission

A combination prevention scenario was developed to investigate using a combination of changes to PrEP uptake, screening rates, time to diagnosis and time to treatment parameters, but without adjusting the use of TasP (Table 3). The aim of this scenario was to investigate whether a combination approach could be used to reach zero transmission without increasing the UK’s current high rate of ART use and virological suppression (97%). In the combination prevention scenario, the annual rate of PrEP uptake was increased to 0.25%, resulting in the number of MSM using PrEP rising from 4.90% of MSM (27,974 individuals) in 2020 to 5.87% of MSM (36,456 individuals) in 2030 (Supplementary Fig. 3).

Under the combination prevention scenario, zero transmission was not reached within the model time horizon (Table 4). The lifetime risk for HIV-negative MSM not using PrEP to acquire HIV fell from 13.65% in the base case scenario to 9.55% in this future scenario (Table 4).

**Table 4:** Results for the base case scenario and combination prevention scenarios with three different rates of TasP

	Base case^a^	Combination prevention	Combination prevention, 98% TasP	Combination prevention, 99% TasP
Year zero transmission is reached	NR	NR	NR	2030
Additional HIV cases over the model time horizon	105,586	73,166	65,405	56,617
Cases avoided, compared with the base case scenario, over the time horizon	N/A	32,420	40,181	48,969
Lifetime risk of acquiring HIV for HIV-negative MSM not using PrEP	13.65%	9.55%	8.61%	7.53%

**Footnotes**: Model time horizon was 50 years (2020–2070). ^a^TasP was set to 97% in the base case and combination prevention scenarios. MSM: men who have sex with men; NR: not reached; PrEP: pre-exposure prophylaxis; TasP: treatment as prevention

#### The impact of TasP

**Fig. 2:**
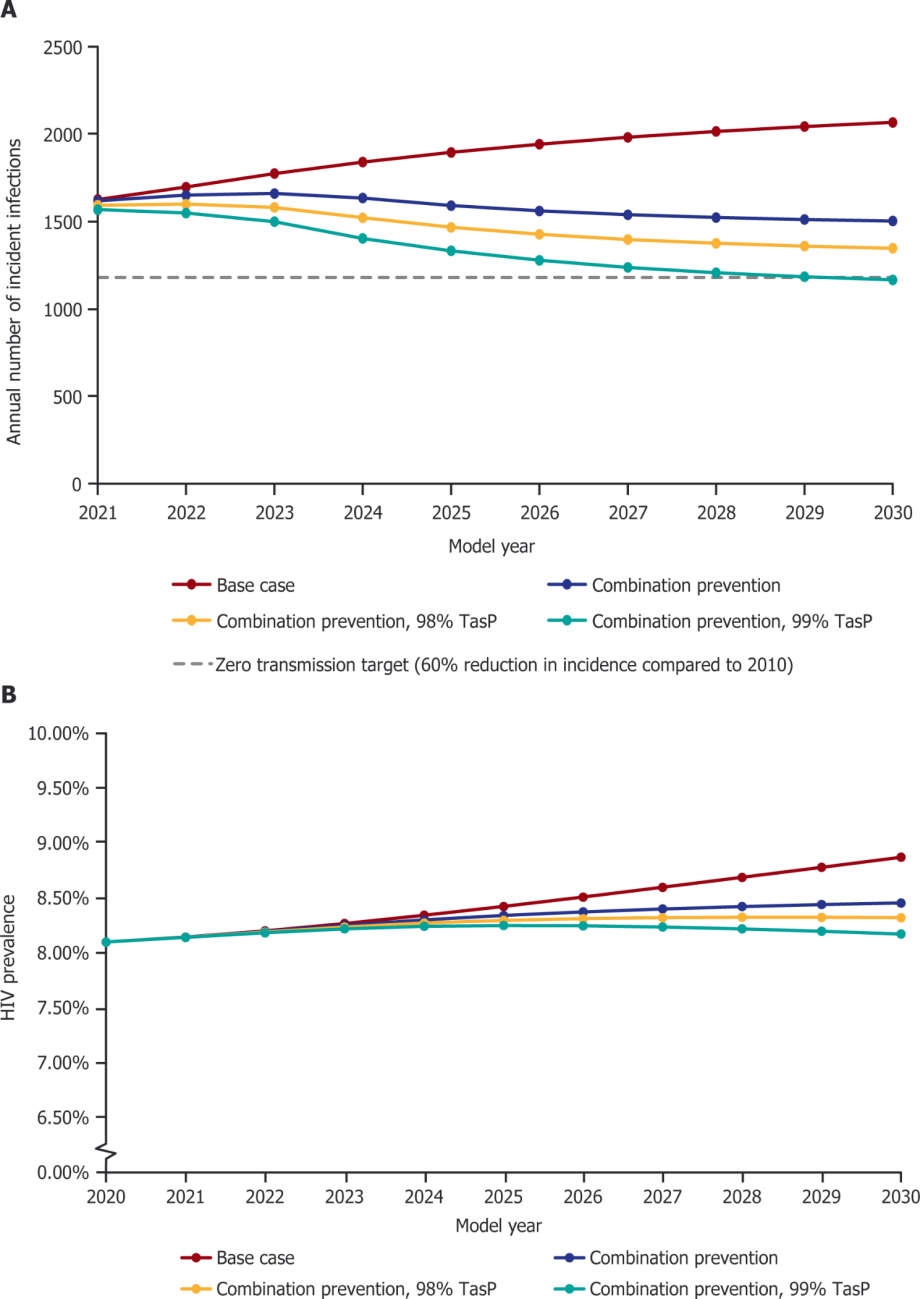
Impact of a combination approach to prevention on HIV incidence (A) and prevalence (B). The incidence reduction target of a 60% decrease in incidence compared with 2010 is marked with a grey dashed line in panel A. TasP: treatment as prevention

The impact of increasing TasP, representing the proportion of MSMLWH on ART who are virologically suppressed and so cannot transmit HIV, was investigated in two additional combination prevention scenarios (combination prevention, 98% TasP and combination prevention, 99% TasP; Tables 3 and 4). Increasing TasP from 97 to 98% did not allow zero transmission to be reached within the time horizon. Increasing TasP to 99% allowed zero transmission to be reached by 2030 resulting in 6,394 additional HIV cases avoided by 2030 and 48,969 additional HIV cases avoided by 2070, compared with the base case scenario (Fig. 2). In addition, the lifetime risk of acquiring HIV for HIV-negative MSM not using PrEP decreased from 13.65% in the base case scenario to 7.53%.

#### Achieving zero transmission before 2030

An aspirational combination prevention scenario was developed to examine when zero transmission could be reached in the model with even greater increases to key parameters. The rate of PrEP uptake, screening rate and probability of starting treatment within three months of diagnosis were set at a higher level than in the combination prevention scenario and TasP was set to 99% (Table 3, Supplementary Fig. 3). These parameters were selected as they are important aspects of the ‘Towards Zero’ action plan [28]. In the aspirational combination prevention scenario, zero transmission was reached by 2026 (Supplementary Fig. 4), resulting in an additional 2,619 HIV cases avoided by 2030 over the combination prevention scenario.

#### Impact of PrEP commissioning

In the second half of 2020, PrEP began to be available to individuals at risk of HIV acquisition through the National Health Service (NHS) in England. Data for the number of MSM accessing PrEP through specialist sexual health services (SHS) in 2021 are now available; [20] these data reveal that the number of MSM accessing PrEP through specialist SHSs is substantially higher than the number that were able to enrol on the Impact trial [20, 21]. To investigate the potential impact of this change in PrEP access through NHS commissioning, the number of MSM using PrEP in the first year of the model was increased to match the number of MSM accessing PrEP through specialist SHSs in 2021 (50,152 MSM; Supplementary Fig. 3) [20] to create the NHS-commissioned PrEP base case scenario; all other inputs remained at their base case values (Table 3). In addition, an NHS-commissioned PrEP combination prevention scenario was developed, bringing together the increased number of MSM on PrEP in the first year of the model and increases to each of the six key parameters in line with the combination prevention scenario (Table 3). The rate of PrEP uptake, i.e. the proportion of HIV-negative MSM initiating PrEP per cycle, in the NHS-commissioned PrEP base case and NHS-commissioned PrEP combination prevention scenarios was not increased from the rates used in the base case and combination prevention scenarios, respectively, as it is not anticipated that the overall rate at which MSM will initiate PrEP in any given year has significantly changed as a result of NHS commissioning. The dramatic increase in the number of MSM using PrEP between 2019 and 2021 can be explained by the change in the availability of PrEP; during this period, free-at-the-point-of-access PrEP became available to all eligible MSM through NHS SHSs, rather than just those eligible MSM who were able to enrol on the Impact trial. Following this change, MSM who were eligible for and interested in PrEP, but not enrolled on the Impact trial, initiated PrEP through SHSs. PrEP uptake is hereafter expected to stabilise, now that the majority of MSM who are considered eligible for the intervention are accessing it [20].

Increasing the number of MSM on PrEP in the first year of the model in the NHS-commissioned PrEP base case scenario resulted in small improvements in the trajectory of the HIV epidemic but did not result in large changes to the number of additional HIV transmissions across the time horizon, or the lifetime risk of acquiring HIV for MSM not on PrEP, compared to the original base case scenario (Table 5).

In the NHS-commissioned PrEP future scenario, 55,558 MSM were using PrEP by 2030 and zero transmission was reached by 2028 (Supplementary Fig. 5), two years earlier than in the original future scenario. In total, 47,009 additional HIV transmissions were avoided over the course of the time horizon in the NHS-commissioned PrEP future scenario compared to the equivalent base case scenario and the lifetime risk of acquiring HIV for MSM not on PrEP fell from 13.20% to 7.28% (Table 5).

**Table 5:** Results for the NHS-commissioned PrEP base case scenario and future scenario

	NHS-commissioned PrEP base case scenario^a^	Future scenario with 2021 PrEP use data and 99% TasP
Year zero transmission is reached	NR	2028
Additional HIV cases over the model time horizon	101,684	54,675
Cases avoided, compared with the base case scenario with 2021 PrEP data, over the time horizon	N/A	47,009
Lifetime risk of acquiring HIV for HIV-negative MSM not using PrEP	13.20%	7.28%

**Footnotes**: Model time horizon is 50 years (2020–2070). ^a^TasP is set to 97% in the base case scenario. HIV: human immunodeficiency virus; MSM: men who have sex with men; N/A: not applicable; NHS: National Health Service; NR: not reached; PrEP: pre-exposure prophylaxis; TasP: treatment as prevention

## Discussion

Having surpassed the UNAIDS 90-90-90 goals, the UK is now targeting zero transmission as the next step towards HIV elimination [4].

The results of this and other modelling approaches suggest the UK may not reach its zero transmission goal without changes to key HIV screening, treatment and prevention policy, [13] however, there remains a limited number of modelling studies that have investigated this topic. The analyses presented here focus on MSM and MSMLWH; in 2019 more HIV diagnoses were made following exposure via sex between men than following exposure via any other route, and MSM represented more than 45% of individuals seen for HIV care in England [7]. Given that MSM represent a key population in the UK who are well engaged with HIV prevention services such as PrEP as well as with HIV care following diagnosis, [20] it is likely that the model’s conclusions around achieving zero transmission in MSM are applicable to the wider population of PLWH in the UK.

The results presented here also show that when prevention, screening or treatment parameters are increased individually, unrealistic scale-ups are required for zero transmission to be reached by 2030. This study demonstrates that a combination approach to HIV policy, rather than individually increasing single prevention, treatment, or screening parameters, is required for ambitious zero transmission goals to be reached in the UK MSM population. These results demonstrate that, once fully implemented, the objectives set out in the UK Government’s ‘Towards Zero’ action plan are likely to be successful in significantly reducing HIV transmission, with major increases in prevention, screening and treatment coverage allowing transmission goals to be reached before 2030.

Screening has been identified by an independent HIV Commission in the UK as ‘the single most important intervention’ for reaching zero transmission by 2030 [22] and the power of increasing screening rates to combat transmission is clearly shown in the model; for example, screening almost all MSM annually would allow zero transmission to be reached by 2030 without changing any other parameters. Increasing HIV screening could occur through several routes. Reducing missed opportunities for HIV testing in sexual health clinics, driven by increased awareness of the importance of testing for epidemic control and education around individual risk of HIV acquisition, is an important factor in improving testing rates highlighted by the ‘Towards Zero’ action plan. Campaigns such as HIV Testing Week are effective at increasing the profile of testing [23] and can also be leveraged to increase testing rates. The role of public awareness in HIV testing is particularly demonstrated by the impact of the 2020 British television drama ‘It’s a Sin’, set in the early days of the epidemic, which was credited with a record number of tests ordered during HIV Testing Week 2021 [24].

PrEP is a vital prevention tool which reduces the susceptibility of individuals at risk of acquiring HIV to HIV transmission, and has been shown to be efficacious in UK and European clinical trials [12, 25]. With PrEP now available through NHS England, the number of MSM known to be using PrEP has risen dramatically and over 70% of MSM identified as having a need for PrEP are now using the intervention. These data also provide the first clear indication of the number of individuals who would be eligible for PrEP in England [20]. Large-scale implementation of PrEP was also identified by Brizzi et al. as a factor likely to be important for reaching zero transmission by 2030 [13]. We therefore investigated scenarios in which the number of MSM accessing PrEP though specialist SHSs in 2021 was implemented as the number of MSM on PrEP in the first year of the model. The results demonstrated that this increase in PrEP use alone was not enough to result in a dramatic change in the trajectory of the HIV epidemic in the UK. This is likely because although the relative increase in the proportion of MSM on PrEP is large (from 24,698 [4.3% of MSM] in 2019 to 50,152 [8.8% of MSM] in 2021), the overall proportion of MSM using PrEP remains at less than 10%. However, when this increase in the initial number of MSM using PrEP was included in a combination prevention scenario, it enabled zero transmission to be achieved within a shorter timeframe.

Downstream from prevention and screening, timely treatment is important for ensuring that diagnosed PLWH are not capable of transmitting the virus. Wider uptake of the RapidART policy used in London’s sexual health clinics, in which individuals start ART within 48 h of diagnosis, [23] and a push to increase the number of people referred into HIV care two weeks after diagnosis [26, 27] should allow ambitious targets for early treatment to be reached.

Finally, the results presented here reinforce the findings of other HIV transmission models in demonstrating the importance of TasP in driving significant reductions in HIV transmission [28]. In addition, these results suggest that a combination approach involving TasP is likely to be a powerful tool for reducing HIV transmission in Western European settings and the USA, due to similarities with the UK in terms of the demographics of populations at risk of HIV transmission, attitudes towards HIV and other social and economic factors. The three combination future scenarios, in which the use of TasP was set to the 2019 level of 97%, or increased to 98% and 99%, show that without increasing the proportion of PLWH who are virologically suppressed from the current rate, the UK is unlikely to reach zero transmission by 2030. This highlights that, as more and more PLWH become aware of their status and start treatment, it is essential that these individuals achieve sustained virologic suppression to prevent the onward transmission of HIV. This may require new policies and programmes to prevent loss-to-follow-up and encourage engagement with care, especially in groups such as the transgender population [29] which present unique challenges.

It is important to note that the combination approach for reducing HIV transmission has wider consequences beyond those who participate in prevention. In the future scenarios explored in our model, the lifetime risk of acquiring HIV for MSM not using PrEP decreased from 13.65% by 4.10–6.12%, depending on the level of TasP modelled. The investment made in a combination approach has wide-ranging population-level impacts beyond the population at risk of HIV acquisition or individuals who engage in HIV prevention, providing positive impacts to individuals at risk of HIV acquisition in hard-to-reach groups with lower engagement in healthcare.

By nature, this model is a simplification of the real world and therefore does not represent all factors associated with HIV transmission. The model was designed to be pragmatic and user-friendly, with the aim of generating discussion around zero transmission and helping to inform decision-making and policy. Thus, the model takes a simplified approach and considers the most important elements of HIV transmission to explore what is needed for accelerating progress towards zero transmission.

The modelling approach presented is a robust model of transmission based on the comprehensive data available for the MSM population in the UK. The model takes a different approach to the work of Brizzi et al. while generating complementary results regarding whether zero transmission can be reached under the current trajectory of the HIV epidemic. The model is also complementary to that of Brizzi et al. in its aim of predicting how changes in screening, treatment and prevention policies can positively impact HIV transmission and make the goal of zero transmission by 2030 attainable. An important difference between the models is the treatment of population growth and migration. Brizzi et al. assumed the MSM population remained constant in size from 2018 onwards and so migration was not accounted for. In contrast, the approach taken here models year-on-year population growth and accounts for non-domestic HIV cases. The addition of incident cases to each model cycle, which cannot be affected by the UK’s domestic HIV policies, necessitates a different approach to defining zero transmission that takes cases occurring outside of the UK into account. However, the zero transmission definition used in our study is broadly aligned with that used by Brizzi et al. and recommended by the HIV Commission [5, 13].

The scenarios presented here do not account for the influence of COVID-19 on HIV transmission in the UK since early 2020. COVID-19-related preventative measures, restrictions imposed on society and impact on healthcare services have had ramifications for individuals’ risk of HIV acquisition and engagement with HIV prevention, screening and treatment, as well as how HIV services are delivered [20, 30]. The significant impact of global COVID-19 policies on population movement, including migration and overseas travel, is also likely to have affected the number of HIV cases identified in the UK that were acquired abroad. In particular, the UK HSA acknowledged issues with data completeness and quality of the 2020 HIV data, as staff shortages and redeployment affected data collection, collation and analysis. Available data showed reductions in both testing and diagnosis rates, as well as a 5% drop in the number of people attending HIV services (either in person or virtually) [30]. These reductions in testing and diagnosis persisted in 2021, while the proportion of PLWH diagnosed late increased, suggesting delays in diagnosis as a result of the pandemic. However, the number of PLWH seen in care in 2021 did increase above 2019 levels [20]. Taken together, these data suggest that the full impact of COVID-19 on HIV in the UK is still not fully understood. Due to the atypical nature of the data collected during the period of the pandemic in the UK, as well as the issues with robustness identified by the HSA, these data have not been used to inform this modelling study. However, as this model does not include the impact of COVID-19 on the transmission of HIV in the UK, predictions regarding the future of the HIV epidemic made in this study may not be fully accurate.

## Conclusions

Based on this analysis in MSM, a key population in the HIV response, the UK is not likely to reach its goal of zero transmission by 2030 without changes to HIV prevention, screening and treatment policies. No single area of HIV clinical or public health policy in isolation offers a silver bullet to substantially reduce HIV transmission without significant, unrealistic scale-ups. This study demonstrates that a combination approach to transmission prevention, as laid out in the UK Government’s ‘Towards Zero’ action plan, has the potential to significantly reduce HIV transmissions in MSM and allow the zero transmission target to be reached. Further research may be required to explore routes to zero transmission in other groups at risk of HIV acquisition such as the heterosexual and transgender populations, where a lack of granular epidemiological and PrEP uptake data currently limits the robustness of any analysis.

### Electronic supplementary material

Below is the link to the electronic supplementary material.

Supplementary Material 1

Supplementary Material 2

## Data Availability

The datasets used and analysed during the current study are available from the corresponding author on reasonable request.

## List of Abbreviations

AIDS: Acquired immunodeficiency syndrome
ART: Antiretroviral therapy
HAS: Health Security Agency
HIV: Human immunodeficiency virus
MSM: Men who have sex with men
MSMLWH: Men who have sex with men living with HIV
NHS: National Health Service
PHE: Public Health England
PrEP: Pre-exposure prophylaxis
PLWH: People living with HIV
SHS: Sexual health services
TasP: Treatment as prevention
UNAIDS: Joint United Nations Programme on HIV/AIDS
U = U: Undetectable equals untransmittable
