# Supplementary material for: Investigating zero transmission of HIV in the MSM population: a UK modelling case study
Source: Arch Public Health. 2023 Nov 20;81:201. doi: 10.1186/s13690-023-01178-0 (PMC10659044; doi:10.1186/s13690-023-01178-0)
Supplement: Supplementary file 1 — Supplementary Material 1 [file 13690_2023_1178_MOESM1_ESM.pdf]

Supplementary Data

Table of Contents

Supplementary Appendix 1: Technical Details of the Zero Transmission Model 2

Model structure 2

HIV Transmission 3

Population age and ageing 5

Population and epidemiology inputs; base case scenario 6

Supplementary Table 1. Epidemiology inputs 6

Supplementary Table 2. Population inputs 6

Implementing changes to epidemiology inputs 7

Disease progression inputs 7

Supplementary Table 3. Hazard ratio for age inputs 7

Supplementary Table 4. Annual disease progression and mortality transition probabilities 7

Transition probabilities 8

Key assumptions 8

Supplementary References 9

Supplementary Appendix 1: Technical Details of the Zero Transmission Model

Model structure

The structure of the Zero Transmission Model is shown in Figure 1 of the main text. The health states illustrated represent the possible 'status' of individuals in the model at any time: individuals can be HIV -positive or -negative, diagnosed or undiagnosed, on treatment or not on treatment, virologically suppressed or unsuppressed. Additionally, HIV-negative men who have sex with men (MSM) are subdivided based on whether they are receiving PrEP. Further, MSM living with HIV (MSMLWH) who are not diagnosed, and MSMLWH who are diagnosed but not on treatment (red and blue boxes, respectively, in Figure 1) are distributed across four health states depending on their CD4 cell count. This allows the model to track the natural progression of the disease if individuals are not treated. MSMLWH who are receiving treatment for HIV are subdivided based on whether they are virologically suppressed or not. On-treatment individuals are assumed to have stable CD4 cell counts over time; individuals who are virologically suppressed are assumed to have a CD4 cell count of >500 cells/mm^3 and individuals who are unsuppressed are assumed to have a CD4 cell count of <200 cells/mm^3.

At each model cycle, HIV-negative MSM not using pre-exposure prophylaxis (PrEP) have both a chance of acquiring HIV and of starting PrEP. Individuals who are 'HIV-negative (on PrEP)' have reduced susceptibility to HIV transmission, based on the efficacy of PrEP. As MSM who are using PrEP are monitored closely and tested regularly for HIV, it is assumed that all MSM who acquired HIV while using PrEP will be diagnosed within three months of transmission (termed the acute phase, a health state lasting for one cycle), whilst in the 'acute phase (on PrEP)' health state.

A proportion of MSMLWH are diagnosed in the 'acute phase' health state, representing the proportion of individuals who are diagnosed within three months of transmission. If not diagnosed in the acute phase, MSMLWH enter the 'HIV-positive and undiagnosed' state, in which they progress through successive CD4 cell count states until they are diagnosed. Individuals who reached the CD4<200 state are diagnosed in the next cycle, as they are detected through their presentation with AIDS-defining illnesses.(1) This assumption was taken from a modelling study in which the model was calibrated against data from a clinical study in Hackney, UK.(1)

When fitting their model, Baggaley et al. (2017) found that values were equivalent to almost instantaneous diagnosis and interpreted as reflecting rapid diagnosis due to high levels of symptomatic presentations.(1)

A proportion of MSMLWH in each undiagnosed health state are diagnosed each cycle, based on the key model variables and model inputs for HIV testing rates. A proportion of these diagnosed individuals move directly to the 'diagnosed and on treatment' state, representative of individuals who start antiretroviral therapy (ART) within three months of their diagnosis. The remainder of the MSMLWH move to the 'diagnosed and not on treatment' state, where they continued to progress through CD4 cell count states until they initiate treatment. At the end of each cycle, 'diagnosed and not on treatment' MSMLWH have a chance to move to one of the 'on treatment' health states.

Individuals that are on treatment are distributed across the 'on treatment and suppressed' and the 'on treatment and unsuppressed' health states every cycle. At the end of every cycle there is a chance that individuals will die, based on the mortality rate associated with their current health state.

New MSM and MSMLWH enter the model each cycle in line with population growth estimates from the Office of National Statistics (ONS).(2) These individuals are distributed across all health states according to the population distribution of the initial model cycle, accounting for the fact that some cases of HIV are known to be acquired outside of the UK.(3)

HIV Transmission

PrEP and ART both reduce HIV transmission. Within the model, the number of individuals who have adopted each of these measures, and their effectiveness, reduces the number of new transmissions each cycle.

- The 'HIV-negative, on PrEP' population has a reduced risk of acquiring HIV (compared with the 'HIV-negative, not on PrEP' population) due to the efficacy of PrEP. PrEP relative reduction in incidence is set to 86% in the model.(4)
- ART reduces an individual's viral load to undetectable levels, which prevents them from transmitting the virus. For individuals who are on ART and suppressed, the reduction in viral transmission due to ART is assumed to be

100%. For simplicity, individuals who are unsuppressed were assumed to be to transmit the virus as readily as individuals not receiving ART.

PrEP and ART effectiveness, and the number of individuals who have adopted these measures, were directly considered in the formula used within the model for calculating the number of new infections that occur each cycle:

$$n\_[\text{new inf}] = \frac{(\beta_0 \times n\_[\text{inf,no ART}] + RR\_ART \times \beta_0 \times n\_[\text{inf,ART}]) \times (n\_[\text{not inf,no PrEP}] + RR\_PrEP \times n\_[\text{not inf,PrEP}])}{n\_[\text{total popn}]}$$

Where:

- *n* is the number of people in a given group,
- inf, no ART is infected not on treatment, and unsuppressed ART MSMLWH,
- inf, ART is HIV-positive, on treatment and suppressed,
- not inf, no PrEP is HIV-negative MSM not receiving PrEP,
- not inf, PrEP is HIV-negative MSM on PrEP,
- *RR_ART* is the relative risk of transmission of persons on ART and suppressed vs. other persons,
- *RR_PrEP* is the relative HIV transmission susceptibility of persons on PrEP vs. other persons,
- Total popn is the number of the total model population (currently alive),
- β is an estimate of the basic reproduction number, that is the number of new HIV cases generated by one MSMLWH (each cycle), if the entire population was susceptible to transmission. The other inputs listed above were employed in the formula to adjust the β factor to take into account the effectiveness of PrEP and ART (which reduce this susceptibility) and the number of individuals that have adopted each measure. Based on the available data, estimating β by rearranging the equation above was deemed the most appropriate approach.

The initial β in the model, i.e. for 2020, was calculated as the average of the computed βs for the years 2012–2018. For each of these years, β was calculated using the same formula described above (rearranged), employing PHE historical data on total MSM population, HIV incidence and prevalence, and the number of MSM

using ART and PrEP. The initial β was used to calculate the number of new transmissions on the second cycle of the model. The estimated number of new transmissions was then used to calculate the β in the following cycle, and so on.

Population age and ageing

UK ONS data were used to inform the starting median age of the HIV-negative population and the annual increase in population median age, which is used to calculate the increase in population median age after each model cycle. This impacts the baseline mortality rate, with older age groups having increased mortality.

It has been observed that, in developed countries, the population of PLWH has a higher median age and that this median age is increasing at a faster rate than the general population.(5, 6) This is because the rate of transmission is decreasing, and because younger people (<35 years old) are less likely to acquire HIV, while people living with HIV are living relatively normal, long lives. It is assumed that this observation around the general population of PLWH also applies to MSMLWH. This was reflected in the model by including a higher median age for MSMLWH, which increased more quickly than the HIV-negative MSM population over time.

Population and epidemiology inputs; base case scenario

Supplementary Table 1. Epidemiology inputs

Input	Value	Source
HIV prevalence	8·10%	Trends in HIV testing, new diagnoses and people receiving HIV-related care in the United Kingdom: data to the end of December 2019 (PHE) – UK data(7)
Proportion of HIV-negative MSM using PrEP	4·90%	Estcourt et al., 2021,(8) PrEP Impact Trial Update – February 2020 (NHS),(9) PHE Expert Guidance
HIV-negative MSM discontinuing PrEP per cycle	1·48%	Coyer et al., 2020(10)
Relative reduction in incidence due to PrEP	86·00%	McCormack et al., 2016(11)
Proportion of MSM tested at sexual health services (proxy for annual probability of screening)	22·35%	HIV in the UK: towards zero HIV transmissions by 2030 (PHE 2019 report)(12)
Annual probability of being screened and diagnosed – MSMLWH with a CD4 count <200	100·00%	Assumption, Baggaley et al., 2017(1)
Proportion of MSMLWH HIV-positive and undiagnosed	5·77%	Trends in HIV testing, new diagnoses and people receiving HIV-related care in the United Kingdom: data to the end of December 2019 (PHE) – UK data(7)
Probability of diagnosis within three months of infection	26·00%	HIV in the UK: towards zero HIV transmissions by 2030 (PHE 2019 report)(12)
ART infectiousness reduction	100·00%	Assumption
Probability of starting treatment within three months of diagnosis	78·00%	HIV in the UK: towards zero transmissions by 2030 (PHE)(12)
Proportion of MSMLWH diagnosed and on treatment	98·44%	National HIV surveillance tables 2020 (PHE)(13)
Probability of starting treatment within six months of diagnosis (CD4 count >200)	95·17%	Assumption based on Prevalence of HIV infection in the UK in 2018 (PHE)(14) ICAR Conference 2019(15)
Probability of starting treatment within six months of diagnosis (CD4 count <200)	98·00%	Croxford et al., 2018(16)
Proportion of MSMLWH with viral load ≤200 copies/mL	97·00%	Trends in HIV testing, new diagnoses and people receiving HIV-related care in the United Kingdom: data to the end of December 2019 (PHE) – UK data(7)
Proportion of untreated MSMLWH with CD4≥500	45·80%	National HIV surveillance tables 2020 (PHE)(13)
Proportion of untreated MSMLWH with 500>CD4≥350	19·06%	National HIV surveillance tables 2020 (PHE)(13)
Proportion of untreated MSMLWH with 350>CD4≥200	1·00%	Calculated
Proportion of untreated MSMLWH with CD4 <200	34·10%	National HIV surveillance tables 2020 (PHE)(13)

Footnotes: ART: antiretroviral therapy; MSM: men who have sex with men; MSMLWH: men who have sex with men living with HIV; NHS: National Health Service; ONS: Office for National Statistics; PHE: Public Health England; PrEP: pre-exposure prophylaxis. CD4 counts are in cells/µL.

Supplementary Table 2. Population inputs

Model input	Value	Source
Total UK population	66,800,000	ONS 2018(2)
Proportion of total population who are MSM	0.93%	BASHH 2020(17)
Total UK MSM population	621,210	Calculated
Males (proportion of the population)	100%	N/A
Median age of the HIV-negative population at model start (years)	40	ONS 2020(18)
Median age of the HIV-negative population at model start (years)	46	Smit et al., 2017(6)
Average population growth per year	0.35%	ONS National population projections: 2018-based(2)
Average increase in median age per year (general population)	0.114	ONS 2019(19)

Model input	Value	Source
Average increase in median age per year (infected population)	0.58	Smit et al., 2015(5) & Smit et al., 2017(6)

Footnotes: BASHH: British Association for Sexual Health and HIV; MSM: men who have sex with men; N/A: not applicable; ONS: Office for National Statistics.

Implementing changes to epidemiology inputs

For key, user-adjustable input parameters, changes are implemented linearly between 2020 to 2024, except for the rate of PrEP uptake, for which changes were implemented linearly between 2020 and 2022. This difference in the rate of parameter change aims to reflect the current status of HIV policy in the UK. The slower increase in rate of screening, time to treatment and TasP parameters aims to reflect the 'lag time' which might be experienced following the implementation of policy change, between the change in policy and full implementation and uptake. In contrast, given that PrEP is now available on the NHS and the level of awareness of PrEP in the MSM community, it is anticipated that PrEP uptake will occur more quickly than other parameters.

Disease progression inputs

Supplementary Table 3 and Supplementary Table 4 outline the disease progression and mortality probabilities used in the model.

Supplementary Table 3. Hazard ratio for age inputs

Age group (years)	Disease progression	Mortality	Source
15–24 (reference)	1	1	Mangal et al., 2017(20)
25–34	1.01	1.25	
35–44	0.94	1.55	
≥45	0.93	2.22	

Supplementary Table 4. Annual disease progression and mortality transition probabilities

From	To	Male 15–24	Source
CD4>500	500>CD4>350	0.205	Mangal et al., 2017(20)
500>CD4>350	350>CD4>250	0.341	
350>CD4>250	250>CD4>200	0.348	
250>CD4>200	200>CD4>100	0.527	
200>CD4>100	100>CD4>50	0.593	
100>CD4>50	CD4<50	0.897	
CD4>500	Death	0.001	
500>CD4>350	Death	0.003	
350>CD4>250	Death	0.003	
250>CD3>200	Death	0.005	
200>CD4>100	Death	0.008	
100>CD4>50	Death	0.014	
CD4<50	Death	0.315	

Footnotes: CD4 counts are in cells/μL.

Transition probabilities

The inputs described previously, plus published data on HIV progression and mortality, informed the probabilities employed in the model (converted as explained above, when needed).

For mortality:

- HIV mortality data by CD4 count(20) informed the chance of moving to the "death" health state from each of these CD4-related health states, regardless of whether individuals in that CD4 count health state are diagnosed or not.
- MSMLWH on treatment who achieved virological suppression had the same chance of dying as HIV-negative MSM (i.e. baseline mortality). Risk of death depends on age such that HIV-negative MSM have a slightly lower rate of death overall than virologically suppressed MSMLWH.
- MSMLWH on treatment who did not achieve virological suppression had the same chance of dying as people diagnosed with AIDS.

For HIV progression:

- The HIV progression probabilities from Mangal et al., 2017(20) informed the transition of diagnosed and undiagnosed MSMLWH to health states with a lower CD4 count, unless they were already CD4<200 (and unless they were diagnosed/started treatment, in which case they transitioned to the corresponding diagnosed/on treatment health states).

Key assumptions

This model is by nature a simplification of the real world. Several assumptions have been made in the model, for example due to insufficient data being available for parameters. The following key modelling assumption was externally validated:

- HIV testing is 100% accurate, with all HIV-positive MSM being diagnosed upon screening. This is a reasonable assumption as fourth generation point-of-care blood tests are standard in the UK and have 99·99% sensitivity and specificity across all subgroups, and all positive HIV tests are followed up with a confirmatory second test to provide diagnostic certainty.

Supplementary References

1. Baggaley RF, Irvine MA, Leber W, Cambiano V, Figueroa J, McMullen H, et al. Cost-effectiveness of screening for HIV in primary care: a health economics modelling analysis. The lancet HIV. 2017;4(10):e465-e74.
2. Office for National Statistics. National population projections: 2018-based. https://www.ons.gov.uk/peoplepopulationandcommunity/populationandmigration/populationprojections/bulletins/nationalpopulationprojections/2018based. Accessed 29 July 2020. 2019.
3. Public Health England. HIV: annual data tables. https://webarchive.nationalarchives.gov.uk/ukgwa/20201015015955/https://www.gov.uk/government/statistics/hiv-annual-data-tables. Accessed 19 July 2022. 2019.
4. Cambiano V, Miners A, Dunn D, McCormack S, Ong KJ, Gill ON, et al. Cost-effectiveness of pre-exposure prophylaxis for HIV prevention in men who have sex with men in the UK: a modelling study and health economic evaluation. The Lancet Infectious diseases. 2018;18(1):85-94.
5. Smit M, Brinkman K, Geerlings S, Smit C, Thyagarajan K, Sighem A, et al. Future challenges for clinical care of an ageing population infected with HIV: a modelling study. The Lancet Infectious diseases. 2015;15(7):810-8.
6. Smit M, Cassidy R, Cozzi-Lepri A, Quiros-Roldan E, Girardi E, Mammone A, et al. Projections of non-communicable disease and health care costs among HIV-positive persons in Italy and the U.S.A.: A modelling study. PloS one. 2017;12(10):e0186638.
7. Public Health England. Trends in HIV testing, new diagnoses and people receiving HIV-related care in the United Kingdom: data to the end of December 2019. https://assets.publishing.service.gov.uk/government/uploads/system/uploads/attachment_data/file/959330/hpr2020_hiv19.pdf. Accessed 09 March 2021. 2020.
8. Estcourt C, Yeung A, Nandwani R, Goldberg D, Cullen B, Steedman N, et al. Population-level effectiveness of a national HIV preexposure prophylaxis programme in MSM. AIDS. 2021;35(4):665-73.
9. National Health Service. PrEP Impact Trial Update - February 2020. Available at https://www.england.nhs.uk/commissioning/spec-services/npc-crg/blood-and-infection-group-f/f03/prep-trial-updates/#February (accessed 10 March 2021) 2020 [
10. Coyer L, van den Elshout MAM, Achterbergh RCA, Matser A, Schim van der Loeff MF, Davidovich U, et al. Understanding pre-exposure prophylaxis (PrEP) regimen use: Switching and discontinuing daily and event-driven PrEP among men who have sex with men. EClinicalMedicine. 2020;29-30:100650.
11. McCormack S, Dunn DT, Desai M, Dolling DI, Gafos M, Gilson R, et al. Pre-exposure prophylaxis to prevent the acquisition of HIV-1 infection (PROUD): effectiveness results from the pilot phase of a pragmatic open-label randomised trial. Lancet (London, England). 2016;387(10013):53-60.
12. Public Health England. HIV in the UK: towards zero HIV transmissions by 2030. https://assets.publishing.service.gov.uk/government/uploads/system/uploads/attachment_data/file/965765/HIV_in_the_UK_2019_towards_zero_HIV_transmissions_by_2030.pdf. Accessed 29 July 2021. 2020.
13. Public Health England. HIV: annual data tables, 2019 data. https://webarchive.nationalarchives.gov.uk/ukgwa/20201229171701/https://www.gov.uk/government/statistics/hiv-annual-data-tables. Accessed 19 July 2022. 2020.
14. Public Health England. Prevalence of HIV infection in the UK in 2018. Health Protection Report. Available at

https://assets.publishing.service.gov.uk/government/uploads/system/uploads/attachment_data/file/843766/hpr3919_hiv18.pdf (accessed 29 July 2020). 2019.
15. d'Arminio Monforte AT, A. Cozzi-Lepri, A. Castagna, S. Passerini, D. Francisci, A. Saracino, F. Maggiolo, G. Lapadula, E. Girardi, C.F. Perno, A. Antinori for the Icona Foundation Study Group. Is time from HIV diagnosis to ART initiation predictive of virological outcome and retention in care? Presented at ICAR 2019. 2019.
16. Croxford S, Yin Z, Burns F, Copas A, Town K, Desai S, et al. Linkage to HIV care following diagnosis in the WHO European Region: A systematic review and meta-analysis, 2006-2017. PloS one. 2018;13(2):e0192403.
17. BASHH. UK Guideline for the use of HIV Post-Exposure Prophylaxis Following Sexual and Occupational Exposures 2020. https://www.bashhguidelines.org/media/1243/pep-2020-consultation-version.pdf. Accessed 11 March 2021. 2020.
18. Office for National Statistics. Population estimates for the UK, England and Wales, Scotland and Northern Ireland: mid-2019. Available at https://www.ons.gov.uk/peoplepopulationandcommunity/populationandmigration/populationestimates/bulletins/annualmidyearpopulationestimates/mid2019estimates (accessed 29 September 2020). 2020.
19. Office for National Statistics. Principle projection - UK summary dataset. Available at https://www.ons.gov.uk/peoplepopulationandcommunity/populationandmigration/populationprojections/datasets/tablea11principalprojectionuksummary (accessed 29 September 2020). 2019.
20. Mangal TD. Joint estimation of CD4+ cell progression and survival in untreated individuals with HIV-1 infection. AIDS (London, England). 2017;31(8):1073-82.
