# Supplementary material for: Investigating zero transmission of HIV in the MSM population: a UK modelling case study
Source: Arch Public Health. 2023 Nov 20;81:201. doi: 10.1186/s13690-023-01178-0 (PMC10659044; doi:10.1186/s13690-023-01178-0)

Supplementary Figures

Supplementary Figure 1. Number of MSM using PrEP over the model time horizon, base case scenario

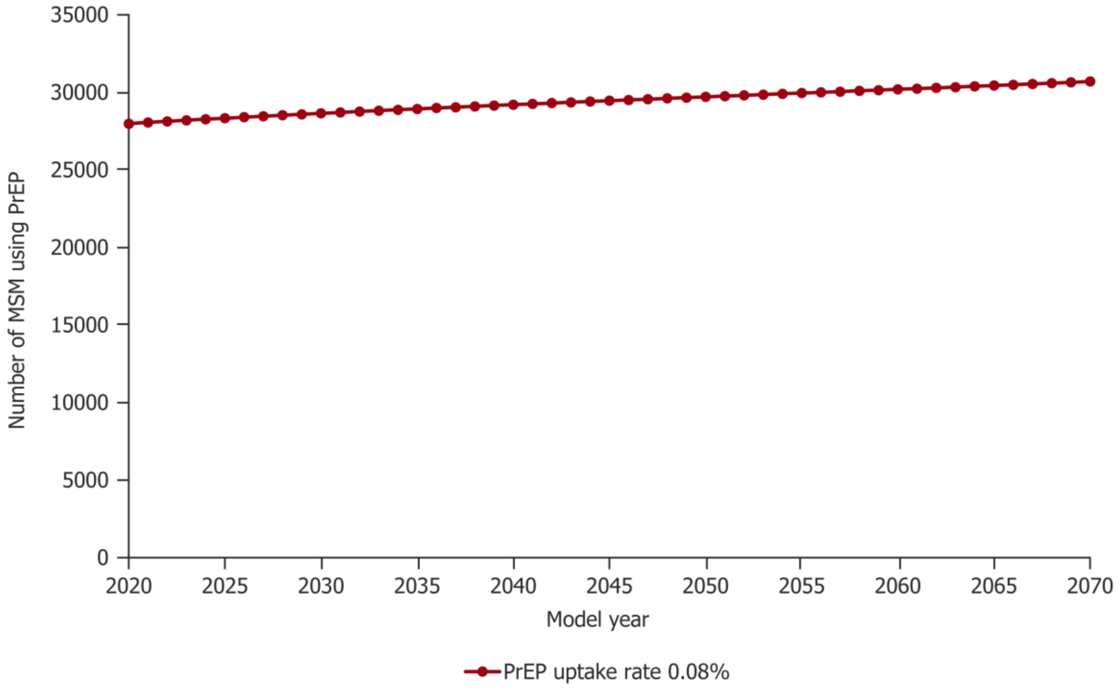

Supplementary Figure 2. Number of MSM using PrEP at two different rates of PrEP uptake, 2020–2030

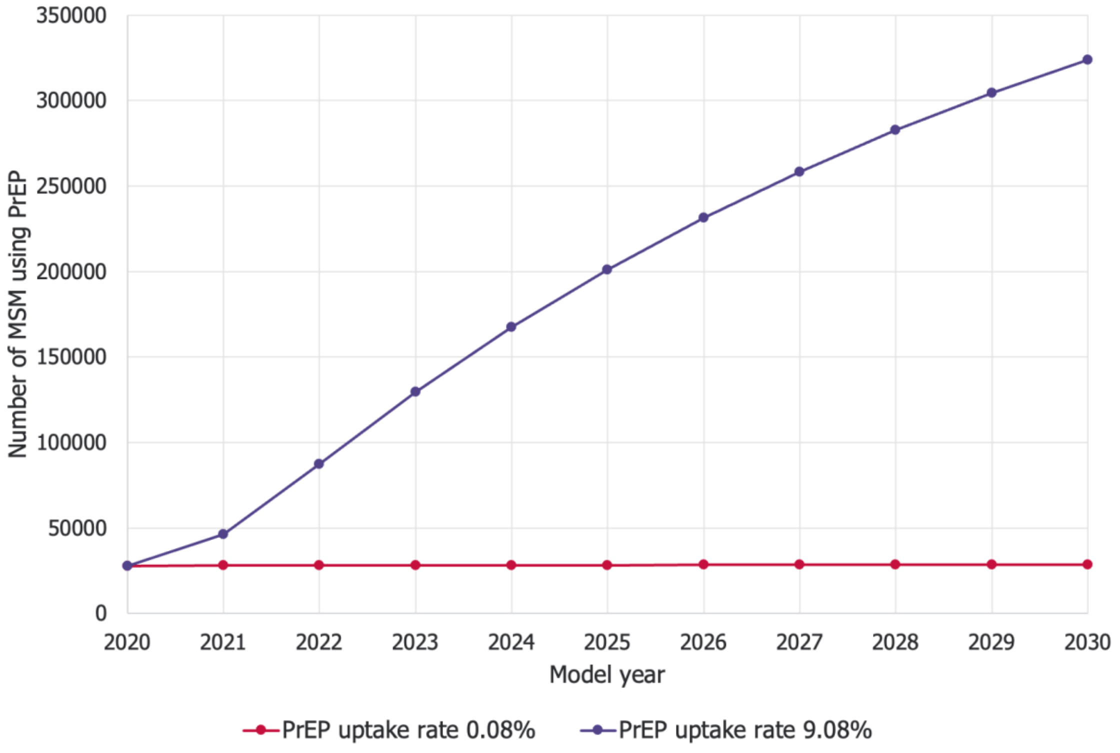

Supplementary Figure 3. Number of MSM using PrEP across all PrEP uptake rates and starting number of MSM using PrEP, 2020–2030

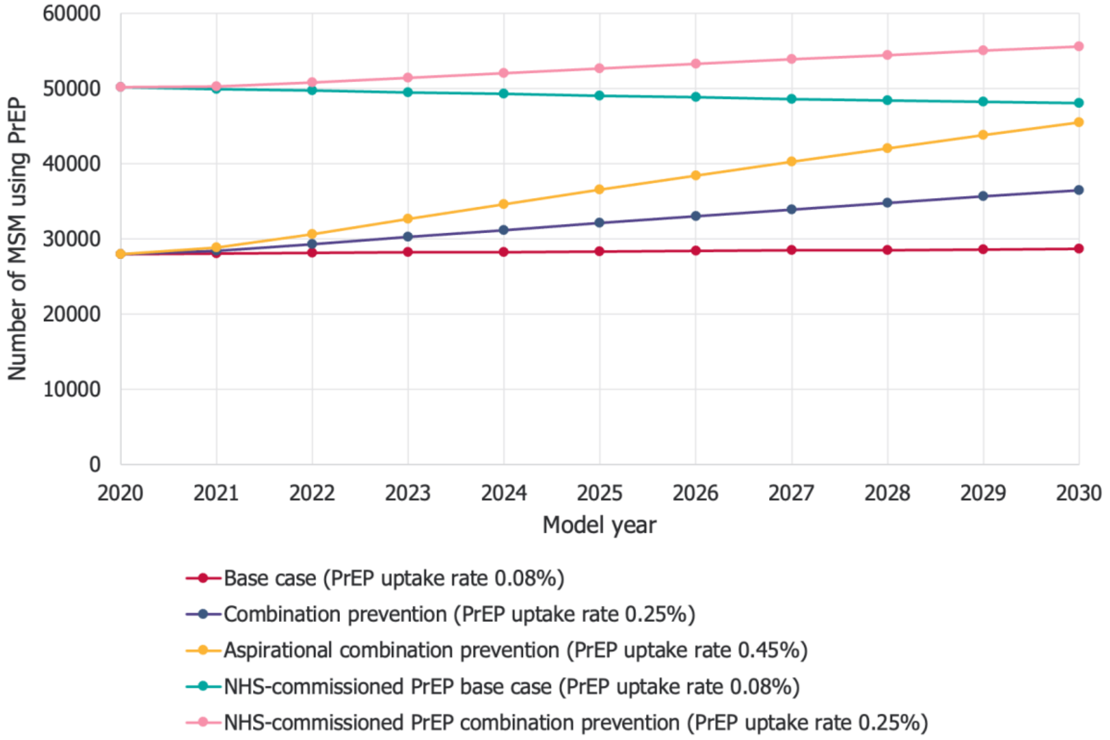

Supplementary Figure 4. Impact of the aspirational combination prevention scenario on HIV incidence

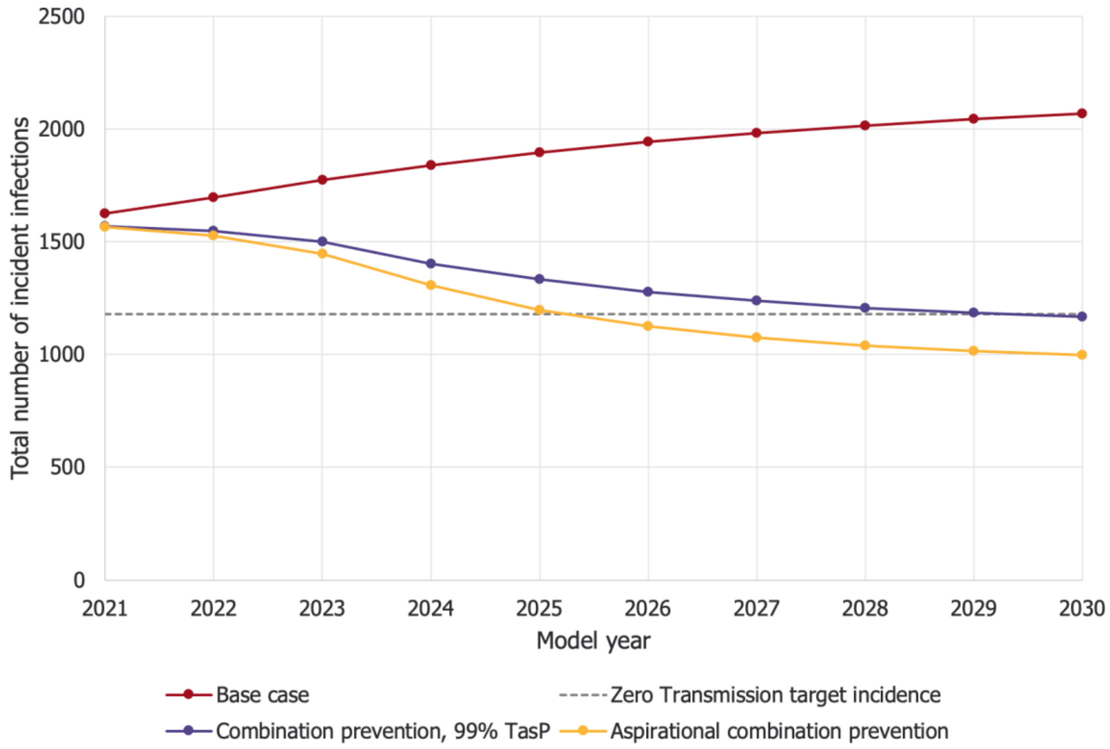

Supplementary Figure 5. Impact of the NHS-commissioned PrEP scenarios on HIV incidence

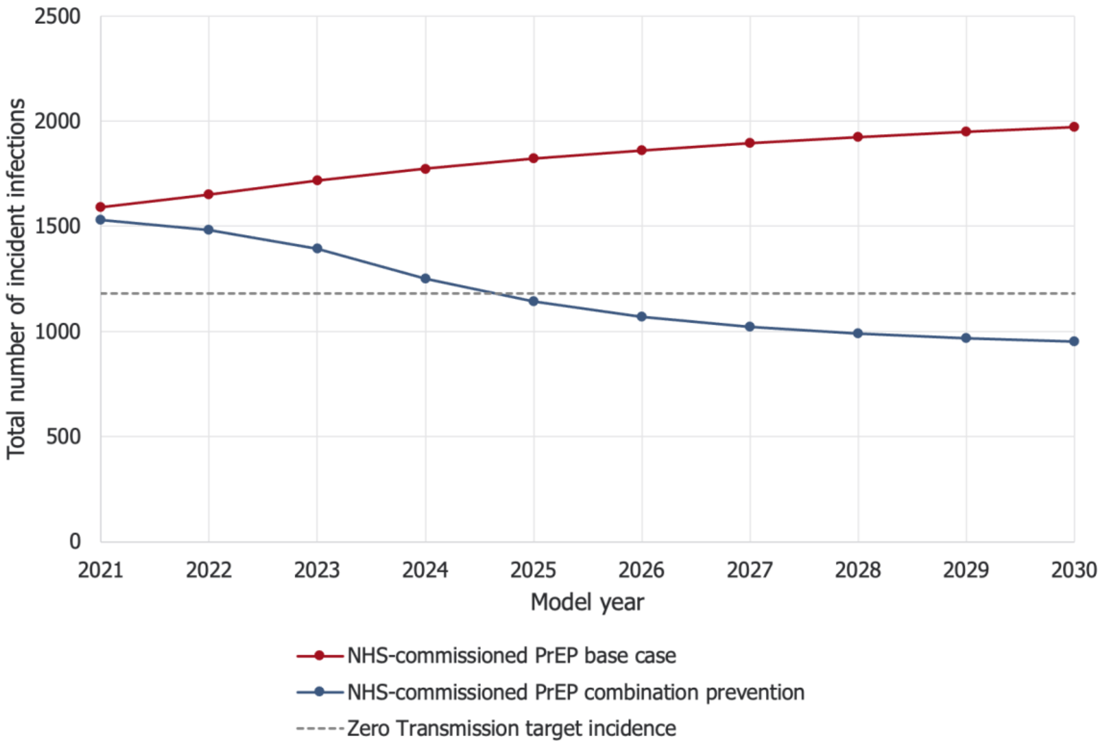

Supplement: Supplementary file 2 — Supplementary Material 2 [file 13690_2023_1178_MOESM2_ESM.pdf]
